# Compassion fatigue and transition shock of internship nursing students

**DOI:** 10.3389/fpsyg.2025.1463319

**Published:** 2025-07-04

**Authors:** Zhi Ping Li, Ling Min Kong, Xin Wang, Jing Wen Qin

**Affiliations:** Department of Nursing, Affiliated Hospital of Yangzhou University, Yangzhou University, Yangzhou, Jiangsu, China

**Keywords:** compassion fatigue, transition shock, nursing internship, job burnout, clinical practicum

## Abstract

**Background:**

Transition shock experienced by nursing students during the middle and later stages of their clinical internships can significantly influence their post-graduation career decisions. Compassion fatigue, prevalent among interns, may exacerbate this transition shock. This study aims to investigate the current status of compassion fatigue and transition shock among nursing students in the middle and late phases of their internships and explore the correlation between these two phenomena.

**Methods:**

A cross-sectional study was conducted using convenience sampling to recruit 201 nursing interns from a tertiary a hospital between July 2022 and June 2023. Data were collected using the General Information Questionnaire, the Professional Quality of Life Scale (ProQOL-5), and the Transition Shock Scale for Undergraduate Nursing Students (UNSTS). Statistical analyses, including univariate analysis and multiple linear regression, were performed to examine the influencing factors of transition shock and the impact of compassion fatigue on transition shock.

**Results:**

The median scores for the three dimensions of compassion fatigue–compassion satisfaction, job burnout, and secondary trauma–were 26 (24, 30), 29 (26, 30), and 28 (25, 30), respectively. The median total score for transition shock was 48 (42, 54). Multiple linear regression analysis revealed that satisfaction with clinical teachers, compassion satisfaction, job burnout and secondary trauma were key influencing factors of transition shock, collectively accounting for 53.2% of its variance.

**Conclusion:**

The findings highlight the severity of compassion fatigue and transition shock among nursing students in the middle and later stages of their internships. Effective mitigation requires structured mentorship, psychological support systems, and resilience training, thereby supporting the retention and professional development of nursing students.

## Background

The clinical internship represents a critical juncture in the professional identity formation of nursing students. The transition from school to clinical practice represents a critical period of role transformation for nursing students. This shift is often accompanied by multiple challenges including high-intensity workloads, role adaptation difficulties, discrepancies between professional expectations and clinical realities, and lack of psychological counseling resources and other factors, it is easy to produce job burnout (Kong et al., 2023). Previous studies showed that only 15.8% of Chinese nursing interns exhibit strong professional identity (Wang et al., 2024) and approximately 5.67% of Chinese final-stage nursing interns expressed explicit dissatisfaction with their vocational choice, while approximately 40% (*n* = 335) reported suboptimal perceptions of the profession’s value proposition (Xie and Han, 2024). This alarming statistic underscores a systemic challenge: the transition from academic training to clinical practice frequently culminates in transition shock–a state of psychological distress stemming from role adaptation difficulties, environmental unfamiliarity, and the theory-practice divide (Rezapour-Mirsaleh and Aghabagheri, 2020; Huang, 2022), posing a critical challenge to the sustainability of the Chinese nursing workforce.

A key yet understudied driver of transition shock is compassion fatigue. Compassion fatigue among nurses often refers to psychological trauma caused by nurses’ long-term exposure to painful situations experienced by patients during the process of providing nursing services (Yu et al., 2018). The Professional Quality of Life Scale-Version 5 (ProQOL-V5) is currently the most widely utilized instrument for assessing compassion fatigue, encompassing evaluations across three critical dimensions: compassion satisfaction, secondary burnout, and traumatic stress (Stamm, 2010). Compassion satisfaction represents the professional fulfillment derived from effective caregiving, serving as a protective factor against excessive psychological distress. Burnout manifests multidimensionally, with emotional exhaustion potentially presenting as persistent irritability, emotional detachment, or chronic fatigue, while physical manifestations may include recurrent headaches and sleep disturbances. Furthermore, reduced professional accomplishment is frequently observed, characterized by self-perceived inadequacies in clinical competence and doubts regarding the value of routine caregiving responsibilities (Wang et al., 2024). Secondary traumatic stress specifically refers to the indirect trauma reactions experienced by helping professionals resulting from prolonged exposure to others’ traumatic experiences. A previous study reported that compassion fatigue also existed among nursing students, especially in their graduation year who were in the transition stage from the school teaching environment to the clinical practice environment (Michalec et al., 2013). Unlike clinical nurses, nursing interns face a dual burden: prolonged exposure to patient suffering while lacking adequate coping resources, coupled with the transitional challenges of shifting from a theory-oriented academic environment to a practice-based clinical setting. Research suggests mid-to-late internship phases (duration > 3 months) represent a high-risk period, where accumulated empathy depletion may impair role adaptation and trigger career attrition (Ko and Kim, 2022). However, few evidence exists on how compassion fatigue mechanistically influences transition shock in this population, leaving educators without actionable insights. This study investigated the prevalence of compassion fatigue and transformation shock among nursing students in the middle and later stages of internships and explored their relationship. Our research findings may provide reference for mitigating transitional shocks, cultivating professional resilience, and ultimately stabilizing nursing talent.

## Materials and methods

### Objective and design

The purpose of this study was to investigate the status of association between compassion fatigue and transition shock among nursing students in the middle and later stages of their internships.

### Participants

The convenience sampling method was adopted during the selection of nursing interns who interned in a tertiary a hospital from July 2022 to June 2023. The inclusion criteria were as follows: ➀ undergraduate or junior college clinical interns majoring in nursing; ➁ internship time exceeding 3 months; ➂ informed consent and voluntary participation in this study. The exclusion criteria was as follows: interns with previous internship experience.

This study adopted a cross-sectional survey design to determine the sample size on the basis of 5–10 times the total number of variables studied. The total number of variables in this study was 18, and 90–180 cases were required for calculation. Considering that 10% of the questionnaires were invalid, 99–198 cases were required. This study ultimately included a sample size of 201 cases, which met the requirements.

### Data collection and questionnaires

The survey questionnaires were distributed via www.wjx.cn, and limitations were set such that an IP address and account could answer only once. The research purpose, precautions for completing the questionnaire, and confidentiality principles were explained before the formal start of the investigation questions. If the students understood and voluntarily participated, they could complete our questionnaires. A preliminary survey was conducted with two nursing students to adjust the language of some items in the questionnaires, and the final survey questionnaire was established before a formal survey was conducted.

### Instruments

#### General information questionnaire

After extensive literature review and discussion by the research group, some general information about the interns was collected, including gender, educational background, whether they voluntarily chose nursing major, whether at least one of their parents was engaged in the medical nursing industry, whether they had experience as a student cadre during their school years, employment direction after graduation, satisfaction with the internship environment and their teachers, and their recent internship duration.

#### Professional quality of life-version 5 (ProQOL-5)

The level of empathy fatigue among nursing interns was evaluated using the Professional Quality of Life Scale, Version 5 (ProQOL-5) (Stamm, 2010), which was translated into Chinese in 2013 (Zheng et al., 2013). The scale is consisted of 30 items and was divided into three dimensions: compassion satisfaction, job burnout, and secondary trauma. Each dimension contains 10 items (1–10, 11–20, and 21–30), and a five-item Likert scale was used to score 1–5 points from “never” to “always.” Among them, five items (1, 4, 15, 17, 29) were scored in reverse, and each dimension was scored separately. The cut off values for the three dimensions were < 37 points, > 27 points, and > 17 points. The lower the compassion score was, the higher the burnout and secondary trauma score, indicating a more severe level of compassion fatigue. The Cronbach’s alphas coefficient of the three dimensions and the overall scale in this study were 0.741, 0.749, 0.638, and 0.882, respectively.

#### Transition Shock Scale for Undergraduate Nursing Students (UNSTS)

The Transition Shock Scale for Undergraduate Nursing Students (UNSTS), which was translated into Chinese in 2022 (Huang, 2022) was used to evaluate the degree of transition shock among nursing interns. The scale had a total of 17 items and six dimensions, including theoretical and practical conflicts, overwhelming internship workload, lack of social support, strained relationships, confusion of nursing professional values, and disharmony between clinical internships and personal life. A 4-item Likert scale was used: 1 = strongly disagree, 2 = disagree, 3 = agree, and 4 = strongly agree with higher total scores indicating greater transition shock intensity. The Cronbach’s alpha of this scale was 0.939 among the 201 interns in the study, and it was 0.819 for a sample of 79 junior college students.

### Statistical analysis

SPSS26.0 was used to analyze the data. After Koimogorov-Smimov testing, the scores of both scales show a non-normal distribution with *P*-values < 0.001. The counting data presented as frequencies and percentages, and the scores of each scale were presented as median and quartile [M, (P25, P75)]. The correlation between the scores of the two scales was analyzed using Spearman’s correlation. Univariate analysis was conducted using the Mann-Whitney *U*-test and Kruskal-Wallis *H*-test. Multiple linear regression analysis was conducted to explore the influencing factors of nursing interns’ transformation impact.

## Results

### Scores of compassion fatigue among nursing interns

The score of the Professional Quality of Life Scale had a non-normal distribution, with a compassion satisfaction score of 26 (24, 30), a job burnout score of 29 (26, 30), and a secondary trauma score of 28 (25, 30).

### Transition shock scores for nursing interns

The scores of the Transition Shock Scale for Undergraduate Nursing Students were not normally distributed, with scores of 8 (7, 9) for theoretical and practical conflicts, 9 (8, 10) for excessive internship workload, and 5 (4, 6) for lack of social support, 7 (6, 9) for interpersonal tension, 13 (11, 15) for confusion in nursing professional values, 5 (4, 6) for disharmony between clinical internships and personal life, with a total score of 48 (42, 54).

### Univariate analysis of the transition shock of nursing interns

The results of the univariate analysis revealed statistically significant differences (*P* < 0.05) in the transformation impact scores across different aspects including education background, voluntary choice of nursing major, employment direction after graduation, satisfaction with the internship environment, and satisfaction with teachers, as shown in Table 1.

**TABLE 1:** Results of the univariate analysis of transition shock among nursing students (*N* =201).

Items	*N*	Total score of transition shock		
		[M, (P25, P75)]	*Z/H*	*P*
**Gender**				
Male	31	48 (40, 54)	−0.079^a^	0.937
Female	170	48 (42, 54)		
**Education**				
Junior college	79	46 (40, 51)	−2.767^a^	0.006
Undergraduate	122	49 (42, 56)		
**Voluntary choice of nursing major**				
No	91	51 (45, 56)	−3.698^a^	< 0.001
Yes	110	46 (40, 51.5)		
**At least one parent is engaged in the medical industry**				
No	195	48 (42, 54)	−0.870^a^	0.384
Yes	6	45 (35.25, 51.75)		
**Student cadre experience during the school**				
No	80	36 (30, 40)	−0.870^a^	0.384
Yes	121	35 (30, 41)		
**Employment direction after graduation**				
Change careers (including cross-major postgraduate entrance examination)	32	54 (47.25, 62.5)	−3.866^a^	< 0.001
Stay in the nursing profession	169	47 (41, 53)		
**Satisfaction with internship environment**				
Very satisfied	36	42 (37.25, 48)	38.129^b^	< 0.001
Satisfied	95	46 (42, 54)		
General	64	51 (45.5, 58)		
Dissatisfied	3	68 (61, –)		
Very dissatisfied	3	65 (63, –)		
**Satisfaction with the teachers**				
Very satisfied	53	42 (38.5, 49)	38.922^b^	< 0.001
Satisfied	101	46 (42, 54)		
General	43	53 (49, 59)		
Dissatisfied	1	–		
Very dissatisfied	3	65 (63, –)		
**Internship duration (month)**				
3–6	159	35 (30, 40)	−1.212^a^	0.226
> 6	42	36.5 (30, 42.25)		

^a^Mann-Whitney *U*-test. ^b^Kruskal-Wallis *H*-test.

### Correlation analysis between compassion fatigue and transition shock among nursing interns

Spearman correlation analysis results revealed that the compassion satisfaction of nursing interns was significantly negatively correlated with the total score of the transition shock and all dimensions of the scale. The score of the occupational burnout dimension was significantly positively correlated with the four dimensions of transition shock. The score of secondary trauma dimension was significantly positively correlated with other dimensions of scores, except for the excessive internship workload of the transformation impact, as shown in Table 2.

**TABLE 2:** Correlation analysis between compassion fatigue and transition shock in nursing students (*N* = 201).

Variables	Compassion satisfaction	Job burnout	Secondary traumatic stress
Theoretical and practical conflicts	−0.294^**^	0.150	0.205^**^
Overwhelming workload	−0.513^**^	0.001	0.041
Lack of social support	−0.553^**^	0.153^**^	0.296^**^
Strained relationships	−0.524^**^	0.178*	0.308^**^
Confusion of nursing professional values	−0.613^**^	0.053^**^	0.177^**^
Incongruity between clinical internship and personal life	−0.551^**^	0.222^**^	0.319^**^
Total	−0.655^**^	0.127	0.271^**^

^**^P < 0.001, *P < 0.05.

### Multiple linear regression analysis of the influencing factors of transition shock among nursing interns

A multiple linear regression model was conducted with the statistically significant variables from the univariate analysis and the scores of the three dimensions of the Professional Quality of Life Scale as the independent variables, and the scores of the Transition Shock Scale as the dependent variables. The results showed that satisfaction with the teachers, compassion satisfaction, job burnout and secondary trauma were the influencing factors for the transition shock. These four variables accounted for 53.2% of the total variation in the transition shock, as shown in Table 3.

**TABLE 3:** Multiple linear regression analysis on influencing factors of transition shock of nursing students (*N* = 201).

Variables	β	*t*	*P*	95% CI	VIF
Constat	–	7,449	< 0.001	(30.888, 53.138)	–
Satisfaction with teachers	0.170	2.432	0.016	(0.385, 3.693)	2.095
Job burnout	0.187	2.146	0.033	(0.038, 0.906)	3.243
Compassion satisfaction	−0.597	−9.453	< 0.001	(−0.908, −0.595)	1.705
Secondary traumatic stress	0.171	2.104	0.037	(0.016, 0.489)	2.836

*R*^2^ = 0.551; Adjusted *R*^2^ = 0.532; F = 29.403; *P* < 0.001.

## Discussion

The results of this study show that nursing interns’ scores for compassion satisfaction, job burnout, and secondary trauma all surpassed critical thresholds, indicating a significant prevalence of compassion fatigue among mid-to-late stage interns., which is consistent with the recent research (Zhou et al., 2013). The observed effects likely stem from compounding stressors: cognitive-emotional overload from sustained patient care exposure (Wu et al., 2021) interacts with competency gaps during escalating clinical demands (Peng and Huang, 2010), creating a stress cascade that manifests as emotional depletion and reduced professional efficacy (Peng and Huang, 2010). Therefore, nursing educators and clinical teachers should incorporate the methods and techniques for emotional regulation into their teaching plans and enhancing communication and feedback mechanisms between instructors and students. At the same time, some targeted intervention strategies can also be adopted based on the characteristics of nursing students, for example, establishing projects for compassion fatigue recovery plan may be an effective way (Pehlivan and Güner, 2020; Li et al., 2023).

Our study found that nursing interns in mid-to-late stages experienced significant transition shock (median score = 48) with “nursing value confusion” being the most severe dimension, which was consistent with the results of a previous study (Wang et al., 2022). This could be due to the significant differences between idealized academic training and clinical realities (Zhang et al., 2021), where interns encounter complex challenges including: procedural tedium, critical patient care, and healthcare conflicts. These experiences might profound cognitive dissonance between the romanticized of “angels in white” image among nursing students, potentially eroding professional identity (Su et al., 2023). Therefore, nursing educators should pay attention to the transition shock status of nursing students in the middle and later stages of internships. Structuring reality preparation during preclinical training and guiding reflective practices to reconcile value conflicts or organizing group psychological counseling (Xu B. et al., 2022) may help our students mitigate these transitional impacts.

This study revealed a significant inverse relationship between satisfaction with clinical instructors and transition shock levels among nursing interns. Instructor satisfaction, a well-established metric for evaluating teaching effectiveness in China (Zhou and Zhu, 2023) serves as a critical determinant of successful clinical adaptation. Higher satisfaction correlates with more comprehensive instructional support, enabling students to acclimate faster to clinical environments, develop enhanced clinical problem-solving competencies (Zhang and Wang, 2021) and experience attenuated transition shock. Empirical evidence identifies two key modifiable factors influencing satisfaction: instructors’ personality characteristics and teaching preparedness (Lim, 2023; Zhang and Hu, 2020). This suggested that healthcare institutions should implement rigorous preceptor selection protocols, optimize teaching management systems, and develop multi-component intervention programs specifically designed to improve instructor-student dynamics and facilitate successful professional transition.

The results of this study indicated that the higher the score of compassion satisfaction, the lower the degree of transition shock of nursing students. In clinical practice, patient recognition of clinical competence, preceptor feedback on skill development, and managerial acknowledgment of professional growth can enhance the professional identity of nursing students (Xu Q. et al., 2022), which is crucial for improving their compassion satisfaction. Clinical nurse educators should adopt an integrated instructional approach that combines deliberate, competency-based training in fundamental bedside procedures under direct supervision with a structured positive reinforcement system delivering behavior-specific feedback to optimize clinical skill development (Hao and Li, 2018), while employing discreet error-correction methods that both preserve learner dignity and uphold professional standards. Crucially, educators must model professional conduct during patient interactions, utilizing teachable moments to demonstrate appropriate clinical decision-making while simultaneously fostering students’ professional credibility.

Our results showed that job burnout could positively affect the transition shock of interns. Unresolved burnout exacerbates transition shock, impairing both role adaptation and professional identity formation. To address this, educators should implement: (1) structured career planning to clarify role expectations, (2) institutional support systems to mitigate stressors, and (3) mindfulness-based interventions to enhance psychological resilience. As a critical dimension of compassion fatigue, secondary trauma refers to healthcare providers’ vicarious experience of patients’ traumatic events, accompanied by distinctive psychological sequelae (Freyberger, 1996). The results of this study showed that secondary trauma among nursing students could positively predict transition shock. Negative psychological experiences induced by occupational exposure will weaken occupational identity and reduce clinical work engagement (Sinclair et al., 2017). This suggests significant professional identity formation challenges during the clinical transition phase. These findings underscore the necessity for educational interventions that concurrently address trauma exposure prevention and psychological aftermath management.

Clinical practicum serves as a pivotal phase in nursing professional development. The persistence of unaddressed burnout symptoms during this transitional period may hinder students’ ability to overcome transition shock, potentially compromising long-term career trajectories (Cao et al., 2021). This study proposes the following interventions for nursing educators to facilitate professional adaptation. The implementation of career planning initiatives can enhance role cognitive restructuring, while strengthening institutional support systems may optimize stressor buffering mechanisms. At the same time, mindfulness-based cognitive interventions should be integrated to cope with the transition shock challenge (Meng et al., 2019). These methods aim to lay a solid foundation for sustainable career development of nursing interns.

## Conclusion

Mid-to-late-stage nursing interns exhibit significant compassion fatigue, which is a key driver of transition shock. We also found that satisfaction with the clinical teachers could also influence students’ transition shock. These findings underscore the urgent need for hospital administrators to implement targeted interventions–such as trauma-informed preceptor training and structured debriefing sessions–to enhance teaching quality and emotional resilience. Nursing educators should integrate transition preparedness modules into curricula and adopt regular compassion fatigue screening using ProQOL-5. Future research should explore longitudinal patterns of transition shock and evaluate digital support tools, while multicenter studies are needed to validate generalizability.

### Limitations

Some limitations of our study can’t be ignored. First, the single-center design restricts the generalizability of findings. Second, it was a cross-sectional design and could not explain the causal relationship between compassion fatigue and transition shock. Third, the marked gender imbalance may bias results toward one gender’s experiences, requiring cautious interpretation of the findings. In the future, multicenter studies involving diverse hospital settings should be conducted to enhance the representativeness of findings. What’s more, longitudinal designs are needed to establish temporal relationships and better understand the causal pathways between compassion fatigue and transition shock.

## Data Availability

The raw data supporting the conclusions of this article will be made available by the authors, without undue reservation.
